# Establishment and Validation of a New Co-Culture for the Evaluation of the Permeability through the Blood–Brain Barrier in Patients with Glioblastoma

**DOI:** 10.3390/pharmaceutics15051431

**Published:** 2023-05-07

**Authors:** Bárbara Sánchez-Dengra, Elena García-Montoya, Isabel González-Álvarez, Marival Bermejo, Marta González-Álvarez

**Affiliations:** Engineering: Pharmacokinetics and Pharmaceutical Technology Area, Miguel Hernandez University, San Juan de Alicante, 03550 Alicante, Spain

**Keywords:** blood–brain barrier (BBB), cell culture, glioblastoma, permeability, in vitro–in vivo correlations (IVIVC), transepithelial electrical resistance (TEER)

## Abstract

Currently, the mechanisms involved in drug access to the central nervous system (CNS) are not completely elucidated, and research efforts to understand the behaviour of the therapeutic agents to access the blood–brain barrier continue with the utmost importance. The aim of this work was the creation and validation of a new in vitro model capable of predicting the in vivo permeability across the blood–brain barrier in the presence of glioblastoma. The selected in vitro method was a cell co-culture model of epithelial cell lines (MDCK and MDCK-MDR1) with a glioblastoma cell line (U87-MG). Several drugs were tested (letrozole, gemcitabine, methotrexate and ganciclovir). Comparison of the proposed in vitro model, MDCK and MDCK-MDR1 co-cultured with U87-MG, and in vivo studies showed a great predictability for each cell line, with R2 values of 0.8917 and 0.8296, respectively. Therefore, both cells lines (MDCK and MDCK-MDR1) are valid for predicting the access of drugs to the CNS in the presence of glioblastoma.

## 1. Introduction

There is great interest in elucidating the mechanisms involved in drug access to the central nervous system (CNS). Much of the effort during drug development is focused on assessing the behaviour of the therapeutic agents with the blood–brain barrier (BBB), including its permeability and the interrelation with the different transport mechanisms. It is widely accepted that the BBB is a dynamic system, capable of responding to local changes and requirements, and able to be regulated via a number of mechanisms and cell types, in both physiological and pathological conditions [1]. This versatile and flexible nature of the BBB is the main drawback in the prediction of drug access to the CNS. Therefore, it does not come as a surprise that many authors put the focus of their attention on the development of models capable of accurately predicting the behaviour of active compounds.

Among the different models that have been proposed in the in vitro assessment of drug permeation across the BBB, the cell culture models have been proven to exhibit great advantages. Cell-based models surmount the main drawbacks of the non-cell in vitro models (Immobilised Artificial Membrane and PAMPA Models); specifically, their absence of transporters and tight junctions. This implies that the cell culture models have the capacity to evaluate the transport mechanisms across the BBB and to mimic its histological physiology. Additionally, pathological conditions can be emulated and, despite the fact that the throughput rate of the cellular models is only moderate, it represents one of the main assets [2].

In light of the origin of the cells (primary or immortalised cell lines), its source (rodent, porcine, bovine and human) and the transporters expressed, numerous cellular models can be proposed, which facilitates the creation of detailed tools for assessing the BBB physiology, pathophysiology and streamlining the drug development process [2,3]. Consequently, all cellular-based models are different, making reproducibility and homogeneity difficult parameters to obtain, notwithstanding the standardisation of protocols and the upswing of immortalised cell lines.

The first cell-based models to appear, as described by Bowman et al. 1983 [4], employed bovine endothelial cells, but it was not until 2003 that Zenker et al. [5] developed a model with enough transepithelial electrical resistance (TEER) value for the prediction of perpetration across the BBB. Subsequently, researchers developed immortalised cell-lines, such as the hCMEC/D3 [6,7] and the Madin–Darby Canine Kidney Cell line (MDCK). The hCMEC/D3 cell line has a human origin and expresses most of the ABCB, ABCC and ABCG transporters. The MDCK cell line has a non-cerebral origin, but it is widely used for its robustness (high TEER values) and its capability for transfection with specific gene transporters, such as the MDR1 gene which expresses the P-gp/ABCB1 transporter. As for today, the models proposed with MDCK cells surpass those with cerebral cells in the in vitro–in vivo correlation of passive compounds, with R^2^ = 0.64 [8] or 0.72 [9] depending on the works consulted. Nevertheless, when evaluating active-transported compounds [8], a non-significant correlation (R^2^ = 0.40) is obtained. As such, the histological differences between MDCK and cerebral lines cannot be overlooked.

In their research on human brain endothelial cells co-cultured with human astrocytes, Megard et al. [10] reported several noteworthy observations: the co-cultures expressed higher TEER values and an upregulation of tight unions and efflux transporters compared to monocultures. These properties, owing to the intercommunication between the endothelial cells and other types of cerebral cells (astrocytes, pericytes, neuroglia and neurons), result in the obtention of an improved in vitro BBB model [3,11].

Because of the obvious advantages, co-cultured models have been satisfactorily used in the past. The co-cultured technique is widely used among different fields. Some examples include: the epithelial–fibroblast co-culture model for the larynx [12], the co-culture model for the evaluation of intestinal absorption [13] and the co-culture model of the lung–blood barrier [14].

The selective impermeability of the BBB is disturbed in infectious (HIV, meningitis, etc.) and/or non-infectious (trauma, hypoxia, tumours, Parkinson’s disease, Alzheimer’s disease, genetic factors, etc.) pathological states. Alteration of BBB integrity varies from mild and transient changes in permeability, resulting from the opening of tight junctions, to chronic disruption of the barrier and irreversible changes in enzyme and transport systems. Increased leakage through the barrier involves microglial activation and infiltration of plasma and immune components into the brain parenchyma, altering the CNS homeostasis and causing variable damage to the normal brain function [15,16,17].

In vitro studies have shown the impact of the glioblastoma in healthy, intact blood–brain barrier models. Mendes et al. [18] proposed a novel in vitro co-culture cell BBB-pathological model using U87 cells (glioma cell line) and immortalised human brain capillary endothelial cells (hCMEC/D3 cell line). In their work, Mendes et al. carried out numerous measurements so as to assess the glioma impact in the barrier. Among them, the TEER measurements and the permeability assays, which employed fluorescein isothiocyanate (FD) of different molecular weights, were the most useful to compare the alterations induced by the glioma cells in the BBB. Mendes et al. noted that, in the presence of the glioma cells, the co-culture expressed lower TEER values and an increased paracellular permeability. Cell morphology alterations were also detected.

Despite the promising results of all the models explored and their feasible application in the assessment of permeability mechanisms of active molecules in healthy and pathological states of the BBB, none of the above-mentioned cell-models can fully predict the pharmacokinetic behaviour of active compounds in vivo [2].

The goal of the present work was to propose a new in vitro blood–brain barrier coculture cell model in the presence of a glioblastoma, which was capable of predicting the in vivo permeability behaviour of different active compounds (letrozole, gemcitabine, methotrexate and ganciclovir). For this purpose, MDCK and MDCK-MDR1 cells were seeded in co-culture with U87 glioma cells. The TEER values and permeability assays were carried out so as to evaluate the difference in permeability between the healthy monoculture model and the glioma co-culture, and to compare the results with the published literature of in vivo studies.

## 2. Materials and Methods

### 2.1. Drugs and Products

The drugs that were tested (letrozole, gemcitabine, methotrexate and ganciclovir) were purchased from Sigma-Aldrich. The MDCK cell line was purchased from ATCC (Manassas, VA, USA), the MDCK-MDR1 cells were provided by Dr. Gottessman, MM (National Institute of Health, Bethesda, MD, USA) and the hCMEC/D3 and the U87-MG cell lines were given by Dr. Sarmento (i3S, Porto). The molecular properties of the drugs that were tested are summarized in Table 1.

The following products were purchased from Sigma-Aldrich: Dulbecco’s Phosphate Buffered Saline (PBS), Dulbecco’s Modified Eagle’s Medium (DMEM), Hank’s balances salt solution (HBSS), Non-essential Amino Acid Solution, HEPES solution, Penicillin–Streptomycin, dimethylsulfoxide (DMSO), basic Fibroblast Growth Factor (bFGF), glutamine, fetal bovine serum (FBS), hydrocortisone, ascorbic acid, trypsin, acid water, acetonitrile (ACN) and fluorescein (4kDa, 40kDa, 70kDa). The Dulbecco’s Modified Eagle’s Medium enriched with pyruvate (DMEM+pyruvate) and the Chemically Defined Lipid Concentrate (CD-LP) were ordered from ThermoFisher and the Endothelial Cell Growth Basal Medium (EBM-2), was bought from Lonza.

### 2.2. Cell Culture and Permeability Studies

MDCK, MDCK-MDR1 and hCMEC/D3 cells lines were used to add the U87-MG cell line and create a new model capable of predicting the in vivo permeability behaviour of different active compounds in patients with glioblastoma. All cells were defrosted and maintained in 75 cm^2^ flasks at 37.5 °C, with a 90% humidity and 5% CO_2_, changing the culture media every other day and sub-culturing them two times a week with a cell density of 6.5·105 cells/cm^2^ for the MDCK, MDCK-MDR1 and hCMEC/D3 cells lines, and 7.5·105 cells/cm^2^ for the U87-MG cell line. This maintenance was conducted for 2 months.

The media used for each cell line was different, according to the characteristic of the cells. The MDCK and the MDCK-MDR1 cell lines were preserved with DMEM, and the glioblastoma tumor line U87-MG was grown with DMEM+pyruvate media. Both mediums were enriched with 1% glutamine, 1% non-essential amino acids, 1%HEPES to maintain the pH of the media, 1% penicillin–streptomycin to avoid the contamination and 10% Fetal Bovine Serum (FBS) to assist the growing of the cells. The hCMEC/D3 cell line was maintained in EBM-2 medium enriched with the same products (FBS, penicillin–streptomycin, HEPES) and CD-LP, hydrocortisone solution and ascorbic acid solution, both filtered with a 0.2 µm filter; in addition, in each flask of hCMEC/D3, bFGF was added at a concentration of 1 ng/mL.

Permeability experiments were carried out on cell monolayers grown on a polycarbonate membrane, 0.4 µm pore size, and with a surface area of 4.2 cm^2^. For this study, the MDCK, MDCK-MDR1 and hCMEC/D3 cells were seeded (6.5 ×10^5^ cells/cm^2^) in the apical side of 6-well transwell plates and they were left to grow until confluence. The confluence was reached in approximately 6–8 days. In order to simulate the glioblastoma illness, on the second day after seeding the MDCK, MDCK-MDR1 and hCMEC/D3 cells, the U87-MG cell line was added (7,5·105 cells/cm^2^) to the basolateral compartment as previously carried out by Mendes et al. [18]. After a week, the confluence of the monolayers was checked by means of measuring the TEER values of each well. If the resistance was good, the experiments were carried out under agitation and at 37 °C, adding the drug solution in the apical chamber and taking samples from the basolateral chamber after 15, 30, 60 and 90 min. With the aim of reproducing the in vivo conditions, the concentrations of the drugs tested were equivalent to the maximum plasma concentration reported in rats, that is, 50 μM for ganciclovir (46.9 μM—[19]), 5 μM for letrozole (3.5 μM—[20]), 50 μM for methotrexate (44 μM—[21]) and 75 μM for gemcitabine (76 μM—[22]). After the last sampling time, cells were washed with HBSS and the TEER value was measured again to test that the membrane was not broken during the experiment due to the drug or the experimental process. After that, the monolayers were disrupted with methanol to recover the drug from inside the cells. Finally, samples were centrifuged and analysed by HPLC or spectrometry.

### 2.3. Analysis of the Samples

Fluorescein, gemcitabine, methotrexate and ganciclovir were analysed by UV spectrometry and letrozole was measured with an UV–HPLC equipment (Waters 2695 separation module, Waters 2487 UV detector and XBridge C18 column (3.5 μM, 4.6 × 100 mm)) using a flow rate of 1 mL/min of a mixture of acid water and acetonitrile (50:50), a run temperature of 30 °C, an injection volume of 90 μL and a run time of 3 min. The wavelengths used for each molecule were: fluorescein 490 nm, gemcitabine 268 nm, methotrexate 258 nm, ganciclovir 252 nm and letrozole 235 nm. All analytical methods were validated and demonstrated to be adequate regarding linearity, accuracy, precision, selectivity and specificity.

### 2.4. Permeability Calculation and Comparison between the Healthy BBB and the BBB with Glioblastoma

The apparent permeability values for the experiments with and without glioblastoma were calculated with the Modified Non-Sink Equation, as conducted before in [23,24] (Equation (1)).

(1) $$C_{r,t}=\frac{Q_{t}}{V_{r}+V_{d}}+\left(\left(C_{r,t-1}\cdot f\right)-\frac{Q_{t}}{V_{r}+V_{d}}\right)\cdot e^{-P_{eff0,1}\cdot S\cdot \left(\frac{1}{V_{r}}+\frac{1}{V_{d}}\right)\cdot \Delta t}$$

In this equation, *C_r_*_,*t*_ and *C_r_*_,*t*−1_ are the concentrations in the basolateral compartment (receiver) at time t and time *t*−1, *V_r_* and *V_d_* represent the volumes of the basolateral (receiver) and apical (donor) compartments, *Q_t_* is the total amount of drug in both chambers at time t, *f* is the sample replacement dilution factor, *S* is the surface area of the monolayer and Δ*t* is the time interval. ***P_eff_***_,0_ is the apparent permeability value at the beginning of the experiment and it can differ from the ***P_eff_*_,_**_1_, which represents the apparent permeability value for the rest of the transport profile.

The Modified Non-Sink Equation was selected for obtaining the permeability values as it proved to be the best option for calculating this parameter, in both sink and no sink conditions, when the initial permeation rate differs from the rest of the transport profile [24]. The initial permeation (***P_eff_*_,0_**) could be lower than ***P_eff_***_,1_ if the partitioning of the drug into the cells was the rate-limiting step and ***P_eff_***_,0_ could be greater than ***P_eff_***_,1_ if, for instance the cell monolayer was affected by a too harsh application of the drug solution. These potential problems made it more appropriate to use the Modified Non-Sink Equation for calculating the permeability values, instead of making just a linear regression as proposed by Artursson et al. with the Sink Corrected equation [24].

The ***P_eff_***_,1_ values, also known as P_app A→B_, were used to compare the healthy BBB model with the BBB+glioblastoma model. For doing this comparison, a ratio between the glioblastoma permeability and the healthy permeability was calculated. If the ratio value was greater than 1, that meant that the in vitro model simulated the disruption provoked in the BBB by the presence of glioblastoma. Subsequently, the in vitro ratios for the different models were compared with the in vivo ones, which were obtained after dividing the AUC in the extracellular fluid of rats with and without glioblastoma; then, several in vitro–in vivo correlations were obtained.

### 2.5. Statistical Analysis

For the statistical treatment, R package version 4.1.3 was used. The differences between the groups were evaluated using a t-student test for independent samples with a statistical significance of *p* < 0.05.

## 3. Results and Discussion

### 3.1. Establishment of the New Co-Culture Models

In 2015, Mendes et al. developed a new in vitro model with the hCMEC/D3 and the U87-MG cell lines which was able to simulate the disruption of the BBB, which is known to be provoked by glioblastoma in vivo [18]. Mendes and collaborators, firstly, checked the permeability of fluorescein with three different molecular weights in the healthy BBB model. The same was undertaken in this study, obtaining the same results.

**Figure 1:**
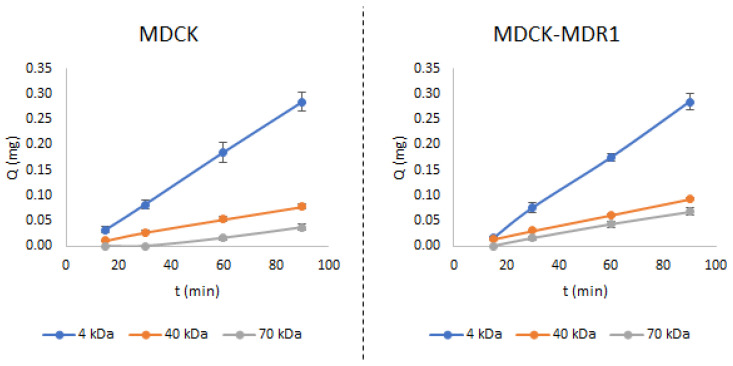
Permeability experiments undertaken with 4 kDa, 40 kDa and 70 kDa fluorescein in the epithelial cell lines MDCK and MDCK-MDR1.

Figure 1 shows that the amount of the drug that was able to cross the BBB was inversely proportional to the size of fluorescein. As such, the 4 kDa fluorescein had the greatest permeability values and the 70 kDa the lowest ones.

**Figure 2:**
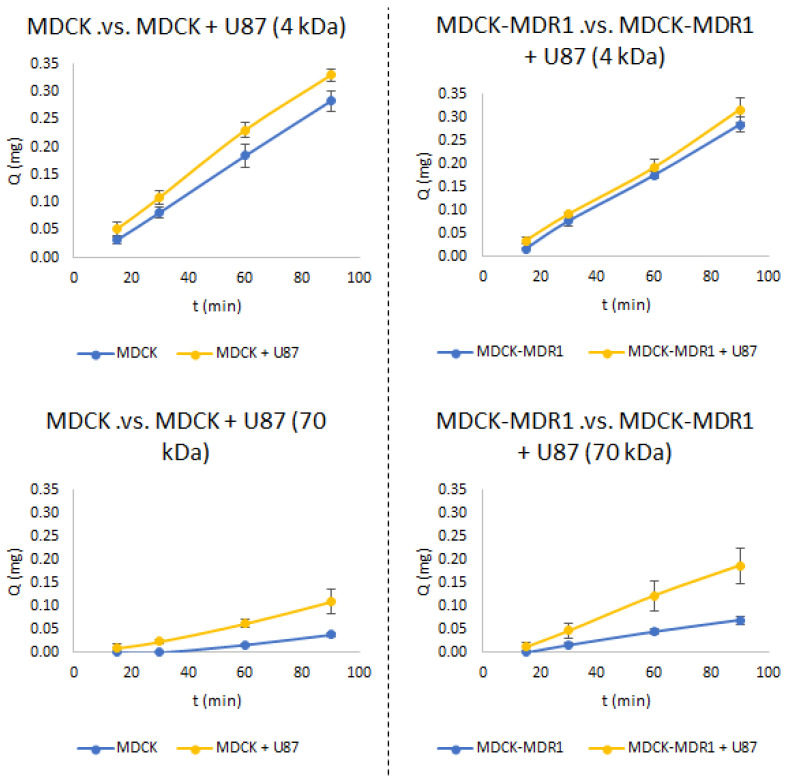
Comparison of the permeability rate obtained with the 4 kDa and the 70 kDa fluorescein in the “healthy” epithelial cell lines MDCK and MDCK-MDR1 and the new glioblastoma co-cultures with the U87-MG cells.

When the same study was repeated, but with combining the MDCK and the MDCK-MDR1 cells with the U87-MG cell line, it was observed that there was a clear increment in the permeation for the fluorescein with the worst access to the CNS when the BBB was intact (the 70 kDa fluorescein) (Figure 2).

There was an increase of 2.74-fold and 2.72-fold in the permeability value of the 70 kDa fluorescein when the U87-MG cell line was added to the MDCK and MDCK-MDR1 monolayers. This increment was observed by Mendes et al. but also by Dwyer et al. using the 40 kDa fluorescein [18,25]. The 4 kDa fluorescein was not affected by the presence of the glioblastoma cells because, due to its small size, it had a high permeability even in the healthy models; the ratios obtained for this molecule were 1.17 and 1.06 for each cell line, MDCK and MDCK-MDR1.

Fluorescein uses a passive access route to cross the BBB and, consequently, no statistically significant differences were observed when comparing the results from the MDCK and the MDCK-MDR1 cells, whose only variance was the extra Pgp transporter in the second ones.

### 3.2. Permeability Studies

Once the behaviour of the new models was checked with the fluorescein molecules and it was considered equivalent to the hCMEC/D3 with U87-MG, they were used to evaluate four different drugs that had been tested in in vivo studies with and without glioblastoma. Contrary to fluorescein, which used the passive diffusion route to access the CNS (as can be extracted from the graphs in Figure 1), the drugs tested used different access routes; the transporters for which they are substrates are summarized in Table 2.

**Figure 3:**
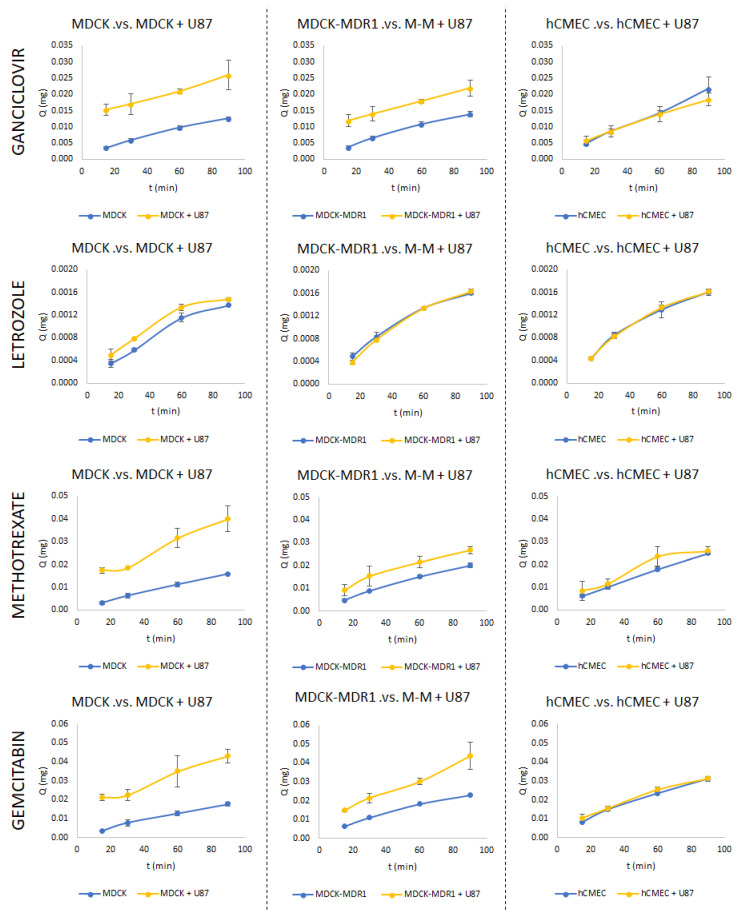
Comparison of the permeability rate obtained with the different drugs in the “healthy” BBB models and the glioblastoma co-cultures with the U87-MG cells.

Figure 3 shows the variation with time of the amount of drug present in the basolateral compartment for all the drugs in the different models (MDCK, MDCK-MDR1 and hCMEC/D3) with and without glioblastoma (U87-MG).

It can be observed that there was a clear increase in the permeation of all the drugs when the U87-MG cells were added to the MDCK and the MDCK-MDR1 models, with the exception of letrozole. Nonetheless, when the hCMEC/D3 experiments were checked, it could be seen that there was not such a clear increment for any of the drugs. Moreover, the “healthy” permeability value was always greater when using the hCMEC/D3 monolayer than with the MDCK and the MDCK-MDR1 cell lines.

These facts can be explained if the properties of each cell line are analysed. The immortalised cell line with human origin hCMEC/D3 (which is considered the gold standard in vitro model for evaluating the access of substances to CNS because it has a lot of BBB transporters), had a TEER value of approximately 30–50 Ω·cm^2^ [7,11]. That means that the monolayers obtained with these cells were not really tightened and they could allow the passage of substances that with other cell lines are prevented from crossing the BBB. The MDCK and MDCK-MDR1 cell lines, which are non-cerebral cells and come from dog kidneys, have higher TEER values [27]. It is because of their higher TEER value that they are also used to simulate the BBB [23,28,29,30], despite their differences with the brain endothelial cell lines [3].

The lower tightness in the hCMEC/D3 cells allows the drugs to more easily cross the BBB and, thus, there is not a relevant increment when adding the glioblastoma cells to the culture. In the other cells, the extra access obtained due to the lack of robustness provoked by the glioblastoma cells can be clearly observed, because the drugs move from being not able to access the CNS to entering the CNS without difficulties.

For letrozole and gemcitabine, drugs which are exclusively the substrate of the Pgp efflux transporter (Table 2), it would be expected that these drugs have a lower access to the basolateral chamber when using the healthy MDCK-MDR1 cell line, due to its extra level of Pgp, but this lower access was not observed (Figure 3). This fact can be explained by the concentrations used to carry out the tests. As previously stated, the concentration selected for the in vitro experiments was the equivalent to the maximum plasma concentration reported in rats and, at that concentration, the transporter may have been saturated and no differences observed between the transfected and the non-transfected cell lines.

Table 3 shows the ratios obtained after dividing the apparent permeability of the BBB+glioblastoma model over the healthy permeability value. The calculation of the ratios showed in a simple way the increment in the permeability observed after adding the glioblastoma cells to the basolateral chamber of a BBB model. These increases can also be observed in Figure 3, comparing the blue (monolayer) and the yellow (co-culture) lines.

### 3.3. In Vitro–In Vivo Correlations

Knowing that the U87-MG cell line was able to alter the behaviour of the different BBB monolayers is interesting, but it is not really useful, unless this alteration can be correlated with what happens in vivo. In that instance, the in vitro studies could be used as a high-throughput screening tool in the development of treatments against glioblastoma.

Having such a type of high-throughput screening tool would save both money and time for the pharmaceutical industries when developing new drugs for the treatment of brain cancer. It is because only the drugs with good access to the CNS in in vitro illness conditions would move to the more expensive in vivo studies.

From an ethical point of view, the co-culture with the glioblastoma cells would also have advantages. For instance, the use of this in vitro model would contribute to the accomplishment of the Rs principles of the use of animals in research (reduction, refinement and replacement). Moreover, less human studies would be needed as the number of failures would be lower.

**Figure 4:**
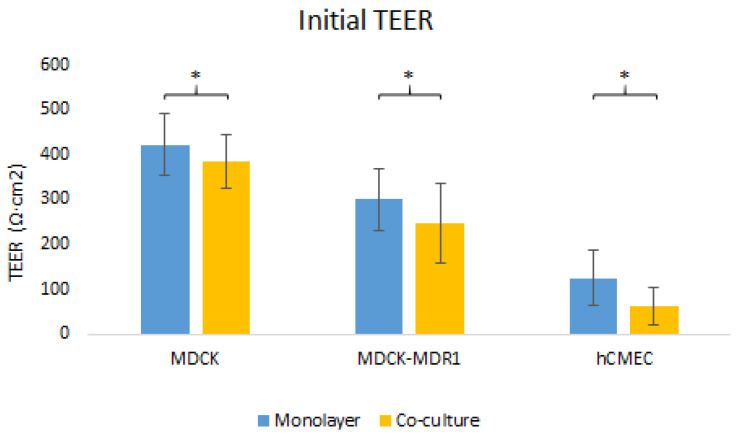
Comparison of the initial TEER values (Ω·cm^2^) between the monolayers and the co-cultures for each cell line studied. * The differences between the three groups were statistically significant (*p* < 0.05).

Figure 4 shows a comparison of the initial TEER values for the three cell lines tested with and without the glioblastoma co-culture. In all cases, the initial TEER value was significantly lower when the U87-MG cells were used. As such, the glioblastoma cells were able to alter the tightness of the BBB.

Four compounds were used for carrying out this study as, when looking at the bibliography, they were the only ones whose biodistribution had been tested in both healthy animals and in animals with glioblastoma.

**Figure 5:**
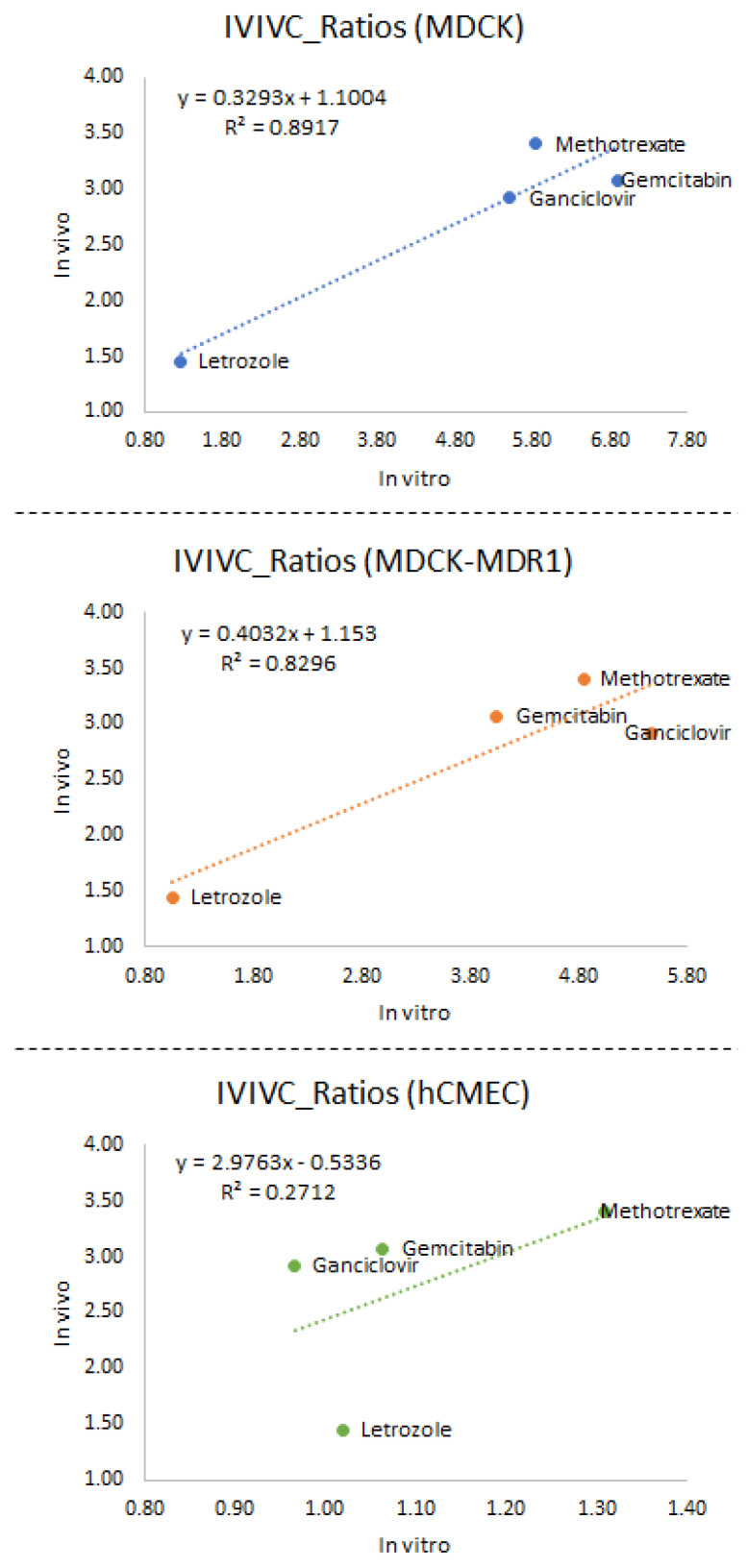
In vitro–in vivo correlations obtained after representing together the ratios obtained with cells and with rats.

The correlations obtained after representing together the in vitro and the in vivo ratios are represented in Figure 5. From these data, it can be extracted that the simplest cell lines (MDCK and MDCK-MDR1) co-cultured with the U87-MG cells reflected in a more reliable way what happens in vivo than the co-culture with the most complex cell line (hCMEC/D3). The coefficient of determination (r^2^) for the correlation with the hCMEC/D3 cells was 0.271, while this coefficient for the other cell lines was greater than 0.800.

This discovery may be surprising, as the hCMEC/D3 cells are considered the gold standard model for evaluating the access of substances to the CNS. However, it must be taken into account that it is the gold standard model when it simulates the healthy BBB. In this case, as it is the disruption of the BBB that needed to be simulated, the low TEER value of the healthy hCMEC/D3 monolayers became disadvantageous as the effect of the glioblastoma cells in the tightness of the monolayers could not be observed.

## 4. Conclusions

The objective of this work was to establish the correlation with in vivo behaviour by establishing a cell co-culture model of epithelial cell lines (MDCK and MDCK-MDR1) with a glioblastoma cell line (U87-MG) that was able to predict the in vivo behavior of compounds. The differences in the TEER values between the monolayer and co-culture models ratified the damage to the integrity of the cell barrier due to the damage to the tumor line. It was seen that use of the U87-MG cell line with the MDCK and MDCK-MDR1 cells gave higher permeabilities than the monolayers models. In addition, the MDCK and MDCK-MDR1 cell lines were valid for predicting the access of drugs to the CNS in the presence of glioblastoma, and had a higher predictive value than the co-culture model with the hCMEC/D3 cell line proposed by other authors. However, to improve the robustness of these conclusions, more studies should be carried out, in vitro and in vivo, to increase the number of compounds included in the correlations.

With the available knowledge, the protocol that the authors recommend following when trying to develop a new tool to treat glioblastoma would be:

(1) Checking the access of the molecule in the MDCK-MDR1 BBB model at different concentrations (one of them being the one selected as effective to kill glioma cells) to evaluate if it is or it is not a substrate of the Pgp transporter.
(2) If the new molecule is a substrate of Pgp, then the access in glioblastoma illness should be evaluated by combining MDCK-MDR1 and U87-MG cells. If the molecule does not bind the Pgp, the permeability should be tested with the MDCK and U87-MG cell lines.

## Figures and Tables

**Table 1:** Molecular properties of the drugs tested.

	MW (g/mol)	Solubility logS (pH 7)	logP	Strongest acidic pKa	Strongest Basic pKa	Charge (pH 7.4)
Ganciclovir	255.23	−1.4	−1.66	10.16	0.58	0
Letrozole	285.30	−3.6	2.50		1.89	0
Methotrexate	454.43	−3.7	−1.85	2.95	14.55	-
Gemcitabin	263.19	−1.1	−1.40	11.52	3.65	0

MW = molecular weight.

**Table 2:** Drugs studied and relevant transporters for which they are substrates [26].

Drug	Transporters
Ganciclovir	-
Letrozole	ABCB1 (P-gp1) *
Methotrexate	ABCC3 *, ABCC4 *, ABCC1 *, ABCC2 *, ABCB1 (P-gp1) *, ABCG2 * SLCO1C1 **, SLC16A1 **, SLC15A1 **
Gemcitabin	ABCB1 (P-gp1) *

* Efflux transporters present in hCMEC/D3; ** Absorption transporters present in hCMEC/D3.

**Table 3:** Glioblastoma/Healthy permeability ratios for each condition tested.

	MDCK	MDCK-MDR1	hCMEC/D3	Rat *
Ganciclovir	5.51	5.47	0.97	2.92
Letrozole	1.28	1.06	1.02	1.45
Methotrexate	5.83	4.85	1.31	3.40
Gemcitabin	6.91	4.05	1.06	3.07

* Rat = AUC_ECFtumor/AUC_ECFbrain.

## Data Availability

Not applicable.
